# Exploring traditional Chinese medicine as a potential treatment for sarcopenia: A network pharmacology and data mining analysis of drug selection and efficacy

**DOI:** 10.1097/MD.0000000000035404

**Published:** 2023-10-13

**Authors:** Changwen Zhou, Hongzhong Ma, Ce Liu, Lixue Yang

**Affiliations:** a The First Clinical Medical Research Institute, Shaanxi University of Chinese Medicine, Shaanxi, China; b Affiliated Hospital of Chinese Medicine, Shaanxi University of Chinese Medicine, Shaanxi, China.

**Keywords:** data mining, network pharmacology, sarcopenia, Traditional Chinese medicine, molecular docking

## Abstract

Sarcopenia, as an increasingly pressing clinical issue, can be ameliorated through employment of traditional Chinese medicines. However, the current lack of specific pharmacological interventions for Sarcopenia necessitates further exploration of novel possibilities in traditional Chinese medicine for the treatment of this condition, utilizing advanced methodologies such as web pharmacology and data mining. Screening the essential targets of Sarcopenia, conducting matching between target and active molecules, as well as active molecules and herbs. Employing data mining techniques to analyze the screening outcomes, and molecular docking to compare the binding activities of active molecules with target proteins. The approach of using herbs for the treatment of Sarcopenia involves 13 targets, with 414 active compounds and 367 types of herbs. Data mining reveals that the herbs used in treating Sarcopenia are primarily characterized by their bitter taste, exerting their effects through dispelling dampness and promoting blood circulation. Moreover, 2 new formulas are postulated. Furthermore, molecular docking analysis indicates that the main active components of the herbs can be observed to tightly bind with the targets. Through network pharmacology and molecular docking, our findings reveal that herbs contain 15 key active components and 5 key targets, which correspond to 7 major herbs and 2 new formulas. Academically, these findings hold significant reference value for the development of novel drugs targeting Sarcopenia.

## 1. Introduction

As a predominant tissue constituting 40% of the total human body weight, skeletal muscle plays multifaceted roles in human physiology, encompassing functions such as movement, metabolism, amino acid storage, and release, and serves as a crucial component in disease prevention and maintenance of overall health^[1]^ as well. Despite the well-known fact that skeletal muscle mass and regenerative capacity decline with aging,^[2]^ the increasing prevalence of skeletal muscle abnormalities in a large population of aging individuals poses a significant health risk in the future. Surveys from Europe, China, and the UK indicate a decline in skeletal muscle function across different regions, with a trend of occurring at a younger age in patients.^[3–5]^

The term “sarcopenia” has long been used as a specific term to describe the loss of muscle mass and function,^[6]^ but early clinical analysis of sarcopenia often relied on the concept of “muscle wasting” (i.e., low muscle mass).^[7]^ In response to various clinical challenges, the European Working Group on Sarcopenia in Older People introduced an international definition of sarcopenia in 2019, which incorporated muscle function assessment into the diagnostic criteria,^[8]^ thereby enabling a more accurate diagnosis of sarcopenia and facilitating in-depth research.

Resistance exercise is considered an effective intervention to improve muscle strength^[9]^ and quality.^[10]^ However, it should be acknowledged that compliance with resistance exercise may be compromised in clinical sarcopenia patients due to age, underlying diseases, and decreased physical capacity, necessitating more gentle and complementary treatment approaches, of which oral medication is a good example. Unfortunately, despite the improved understanding of sarcopenia, there is currently no specific drug approved for the treatment of sarcopenia. Instead, there is only preliminary evidence suggesting that myostatin inhibition^[7]^ might be potentially beneficial. Therefore, the development of drugs targeting at sarcopenia holds significant clinical significance and warrants further research.

Traditional Chinese medicine (TCM), with a history of thousands of years of clinical application, has accumulated ample clinical experience in the treatment of various diseases. A systematic review has highlighted the favorable therapeutic effects of TCM on sarcopenia.^[11]^ According to theories of TCM, different herbal medicines can interact with each other to achieve better treatment outcomes, and herbal medicines have advantages such as a diverse array of effective compounds and stable compound structures. In this study, the potential of Chinese herbal medicine for treating sarcopenia based on network pharmacology and data mining techniques is explored. Unlike traditional research models which focus on specific Chinese medicine formulas for sarcopenia, this study takes a different approach by investigating what kind of herbs are needed for sarcopenia and matching them with existing formulas to explore novel Chinese medicine formulas for the treatment of sarcopenia.

## 2. Materials and methods

**Figure 1.**
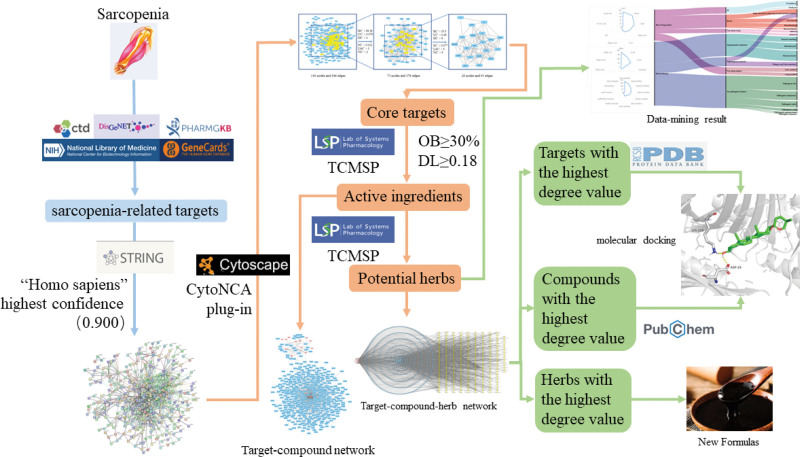
A summary and depiction of the workflow investigating the potential of Chinese herbal medicine for treating Sarcopenia were elaborated. Relevant targets associated with Sarcopenia were downloaded from 5 different databases. A protein-protein interaction (PPI) network was constructed using the STRING database, and core targets were selected using Cytoscape software and the CytoNCA plugin. Active ingredients and herbs were searched in the TCMSP database, and data mining techniques were employed to analyze the herbs. Finally, molecular docking was performed to validate the potential targets of herbs for treating Sarcopenia. DL = drug-likeness, OB = oral bioavailability, PPI = protein-protein interaction, TCMSP = Traditional Chinese Medicine Systems Pharmacology.

The research process is divided into several parts, as illustrated in Figure 1. Firstly, established databases are searched for targets related to Sarcopenia. Subsequently, targets, compounds, and herbal medicines are matched one by one to construct a “targeted pathway” network to analyze potential herbs for treating Sarcopenia. Finally, molecular docking is performed to reveal the interaction patterns between active molecules and target proteins.

### 2.1. Construction of sarcopenia-related targets database

The targets associated with sarcopenia were sourced from 5 databases, including GeneCards databases^[12]^ (https://www.genecards.org/), DisGeNET databases^[13]^ (https://www.disgenet.org/),Comparative Toxicogenomics Database^[14]^ (CTD:http://ctdbase.org/), National Center for Biotechnology Information^[15]^ (NCBI:https://www.ncbi.nlm.nih.gov/), and PharmGKB databases^[16]^ (https://www.pharmgkb.org/). Using the keyword “Sarcopenia” for searching, the retrieved results were merged, deduplicated, and used to establish a database of sarcopenia targets.

### 2.2. PPI network construction and core targets screening

Next step involves importing the data of Sarcopenia targets into the STRING database^[17]^ (https://cn.string-db.org/), selecting “Homo sapiens” in the Organisms panel, and setting the minimum required interaction score to “highest confidence” (0.900). After importing the obtained PPI data into Cytoscape 3.7.2,^[18]^ and analyzing the data using the “CytoNCA” plugin, this study utilizes 6 parameters to filter the core targets, including betweenness centrality, closeness centrality, degree centrality, eigenvector centrality, local average connectivity, and network centrality, where the values of these 6 parameters indicate the importance of the target in the entire network.

### 2.3. Active ingredients screening and target-compound network construction

The core targets into the Traditional Chinese Medicine Systems Pharmacology database^[19]^ (TCMSP: https://tcmsp-e.com/) was imported, and then compounds that can interact with such targets were gathered, and the compounds based on the conditions of oral bioavailability (OB) ≥ 30% and drug-likeness (DL) ≥ 0.18 are screened. After screening, the compounds and targets into Cytoscape 3.7.2 for constructing a target-compound network were imported.

### 2.4. Herb matching and target-compound-herb network construction

The compounds into the TCMSP database were imported, herbs that contain these compounds (considered potential herbs for treating Sarcopenia in this study) were gathered, and a compound-herb network was then constructed. After importing the compound-herb network into Cytoscape 3.7.2, it was merged with the target-compound network to create a target-compound-herb network. Then, the “Network Analyzer” plugin was utilized to analyze the network and identify the key targets, key active components, and major herbs based on their highest degree values.

### 2.5. Characteristics of herbs

Through the utilization of data mining techniques, an analysis of potential herbs is conducted. The analysis encompasses characteristics such as medicinal properties, flavors, channel tropism, and therapeutic effects. In consideration of variations in information sources pertaining to medicinal substances, this research employs a standardized approach, sequentially referencing the “Pharmacopoeia of the People Republic of China,”^[20]^ “Chinese Pharmacy,”^[21]^ “Chinese Materia Medica,”^[22]^ and “Dictionary of Traditional Chinese Medicine.”^[23]^ Major herbs are then arranged and combined, with the data subsequently imported into the “Chinese Medical Classic” formula database^[24]^ for reverse searching to identify new formula combinations.

### 2.6. Molecular docking of the target-compound

To evaluate the credibility of the correlation between the target and the compound, and to identify novel herbs for sarcopenia treatment, molecular docking of the key compounds with key targets was conducted. The top 5 targets, as determined by their prominence in the “target-compound-herb” network, were designated as receptors, while the key compounds served as ligands.

The crystal structures of the 5 proteins were obtained from the Protein Data Bank^[25]^ (PDB: https://www.rcsb.org/) and saved in PDB format. The 3-dimensional conformer structures of the key compounds were retrieved from the PubChem database^[26]^ (https://pubchem.ncbi.nlm.nih.gov/) and converted to PDB format using Chem3D. Ligands and receptors were prepared using AutoDock Tools (v.1.5.6) and PyMOL (v.2.3). The preparation of receptors involved the removal of original ligands and water molecules from the crystal structures of receptors, the addition of nonpolar hydrogens, and the calculation of Gasteiger partial charges. The treatment of ligands included energy minimization and assignment of atomic charges and atoms. All the prepared receptors and ligands were saved in pdbqt format.

Subsequently, the affinity, indicating the binding strength between the ligand and the target protein, was assessed using Autodock Vina (v.1.1.2). Furthermore, Pymol 2.3 was employed for visualizing and analyzing the docked conformations, and the docking results with lower binding energy and superior conformation were selected for presentation based on the binding conformations of each compound.

## 3. Results

### 3.1. Sarcopenia-related target acquisition

By querying the disease database for “Sarcopenia” related targets, specifically using an Inference Score ≥ 10 as a filtering condition in the Comparative Toxicogenomics Database database, a total of 468 disease targets were obtained after integrating and eliminating duplicates.

### 3.2. PPI network construction and screening

**Figure 2.**
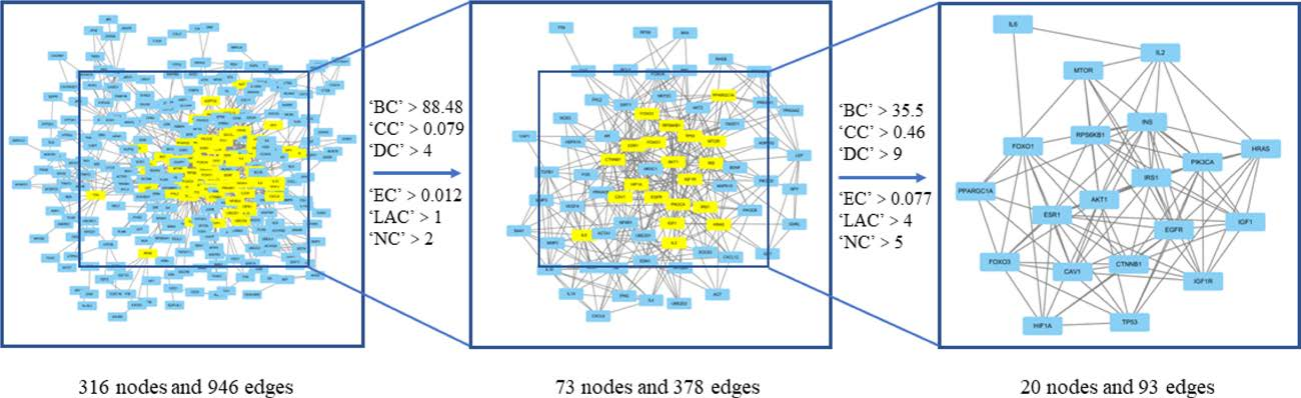
Screening strategy for core targets. Import the PPI data exported from the STRING database into cytoscape, and use the 6 parameters of BC, CC, DC, EC, LAC, and NC calculated by the CytoNCA plug-in to perform 2 screenings. In this study, targets that are greater than the median of the 6 parameters in both screenings are considered as core targets. BC = betweenness centrality, CC = closeness centrality, DC = degree centrality, EC = eigenvector centrality, LAC = local average connectivity, NC = network centrality.

By importing targets into the STRING database to construct a protein-protein interaction (PPI) network consisting of 316 nodes and 946 edges, this study has utilized topological analysis to identify the core network. Two rounds of screening were conducted with the criteria that betweenness centrality, closeness centrality, degree centrality, eigenvector centrality, local average connectivity, and network centrality parameters should all exceed the median, resulting in the identification of 20 core targets as shown in Figure 2. Among these 20 targets, 13 were successfully matched to compounds that met the screening standards of OB ≥ 30% and DL ≥ 0.18 and were thus identified as potential targets for herb treatment of Sarcopenia, as listed in Table 1.

**Table 1:** Information of potential targets.

ID	Gene symbol	Uniprot ID	Protein name
1	MTOR	P42345	Serine/threonine-protein kinase mTOR
2	IL2	P60568	Interleukin-2
3	HIF1A	Q16665	Hypoxia-inducible factor 1-alpha
4	RPS6KB1	P23443	Ribosomal protein S6 kinase beta-1
5	CAV1	Q03135	Caveolin-1
6	ESR1	P03372	Estrogen receptor
7	AKT1	P31749	RAC-alpha serine/threonine-protein kinase
8	CTNNB1	P35222	Catenin beta-1
9	IGF1R	P08069	Insulin-like growth factor 1 receptor
10	IL6	P05231	Interleukin-6
11	FOXO1	Q12778	Forkhead box protein O1
12	EGFR	P00533	Epidermal growth factor receptor
13	TP53	P04637	Cellular tumor antigen p53

### 3.3. Candidate compound acquisition and target-compound network construction

**Figure 3.**
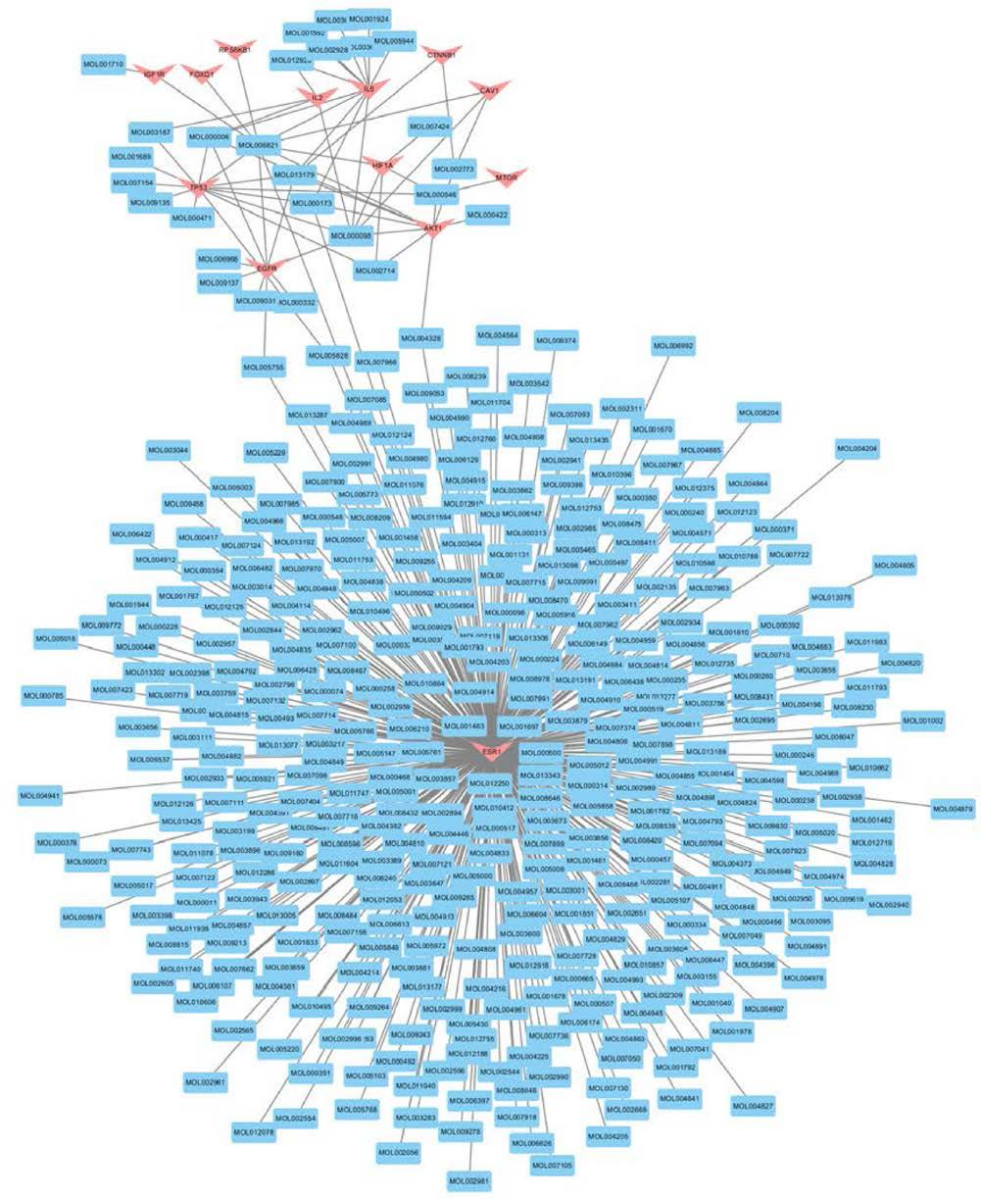
Visualized network of potential target and compound matches. The network consists of 427 nodes and 455 edges. The red nodes in the figure represent targets, the blue nodes represent compounds, and the edges represent the relationship between nodes. The Degree value indicates the number of nodes connecting edges, and the higher the Degree value, the more significant the adjustment effect in the entire network.

By utilizing the TCMSP database, a search for potential targets was conducted and a total of 914 compounds was collected. Among them, 414 compounds met the screening criteria of OB ≥ 30% and DL ≥ 0.18. The target-compound network was constructed based on the potential targets and related compounds, as shown in Figure 3, consisting of 427 nodes and 455 edges. In the network, red nodes represent targets, while blue nodes represent compounds, and edges represent the relationships between adjacent nodes. The Degree value indicates the number of connecting edges of a node, while a higher Degree value indicates a more significant regulatory role in the entire network. The results revealed that ESR1 exhibited the highest Degree value (392) compared to other targets, making it the most pivotal target in herb treatment for Sarcopenia.

### 3.4. Herb acquisition and target-compound-herb network construction

**Figure 4.**
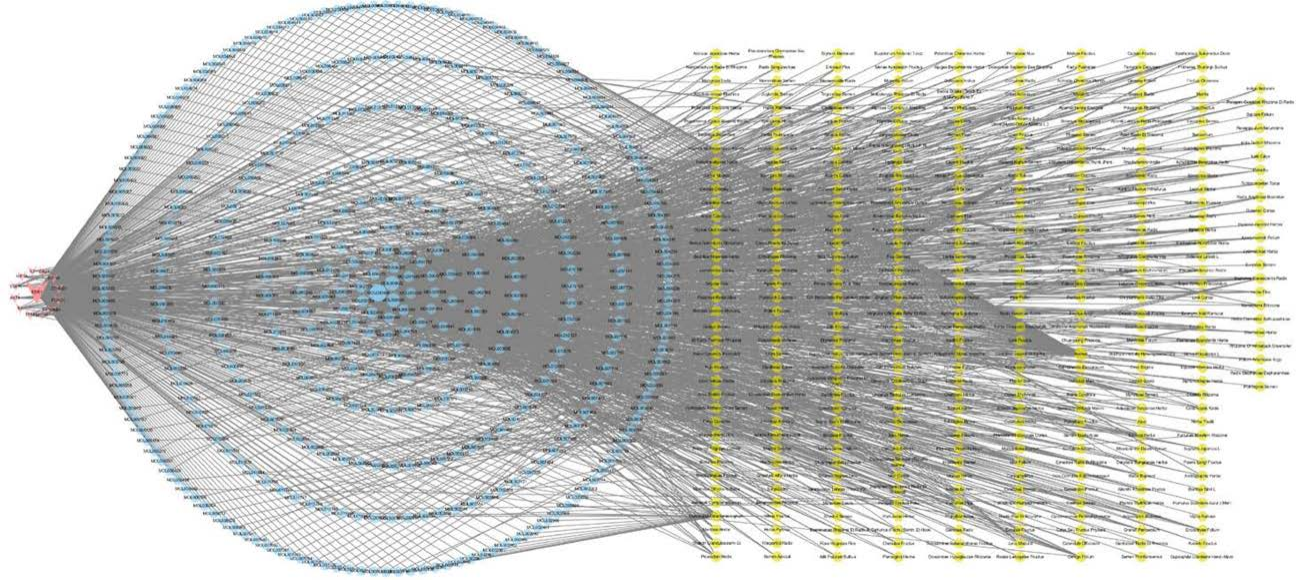
Target-compound-herb visualization network. The network contains 792 nodes and 1789 edges. Red nodes represent targets, blue nodes represent compounds, and yellow nodes represent herb nodes. The size and opacity of each node are positively related to its Degree value.

By matching 414 compounds with 367 herbs in the TCMSP database, a target-compound-herb network was constructed, as shown in Figure 4. This network comprises 792 nodes and 1789 edges, with red nodes representing targets, blue nodes representing compounds, and yellow nodes representing herbs. The size and opacity of each node are positively correlated with its Degree value.

The median Degree value of these targets is 4, and targets with Degree values greater than or equal to twice the median are considered key targets. ESR1, TP53, IL6, AKT1, and EGFR are identified as key targets for treating Sarcopenia with corresponding Degree values of 392, 13, 12, 10, and 9, respectively.

The median Degree value of compounds is 2, but due to insufficient data after 2 rounds of screening based on twice the median, 15 key compounds are supplemented based on their Degree values, as shown in Table 2. Among them, quercetin and kaempferol are compounds with Degree values >100.

**Table 2:** Information of key compounds.

MolID	Mol Name	Degree
MOL000098	quercetin	195
MOL000422	kaempferol	134
MOL000006	luteolin	99
MOL000492	(+)-catechin	42
MOL000354	isorhamnetin	41
MOL002773	beta-carotene	32
MOL000073	ent-Epicatechin	28
MOL004328	naringenin	24
MOL001689	acacetin	22
MOL000546	diosgenin	19
MOL000392	formononetin	19
MOL001002	ellagic acid	18
MOL001040	(2R)-5,7-dihydroxy-2-(4-hydroxyphenyl)chroman-4-one	18
MOL002714	baicalein	15
MOL003044	Chryseriol	15

The top 7 herbs with the highest Degree values are licorice (GanChao), Curcuma aromatica (JiangXiang), Salvia miltiorrhiza (DanShen), Sophora flavescens (KuShen), Corydalis yanhusuo (YanHuSuo), Morus alba cortex (SangBaiPi), and Morus alba folium (SangYe), which are associated with 79, 25, 21, 15, 14, 13, and 12 compounds, respectively, and are considered major herbs.

### 3.5. Properties, tastes, meridian tropism, and therapeutic effects of herbs

**Figure 5.**
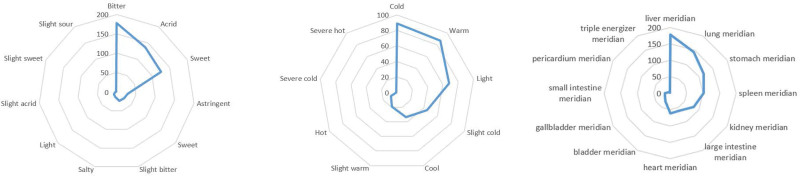
Display of herb characteristics. From left to right are the tastes, properties, and meridian tropism of herbs.

Upon conducting an analysis of properties, tastes, and meridian tropism for 367 potential herbs, the results indicate that the bitter taste is the most commonly occurring feature, followed by acrid and sweet tastes. The combined frequency of these 3 tastes reaches 80.04%. Both cold-natured and warm-natured herbs are commonly found, with frequencies exceeding 24%. In terms of meridian tropism, the liver meridian, lung meridian, spleen meridian, and stomach meridian are the most frequently associated, as depicted in Figure 5.

**Figure 6.**
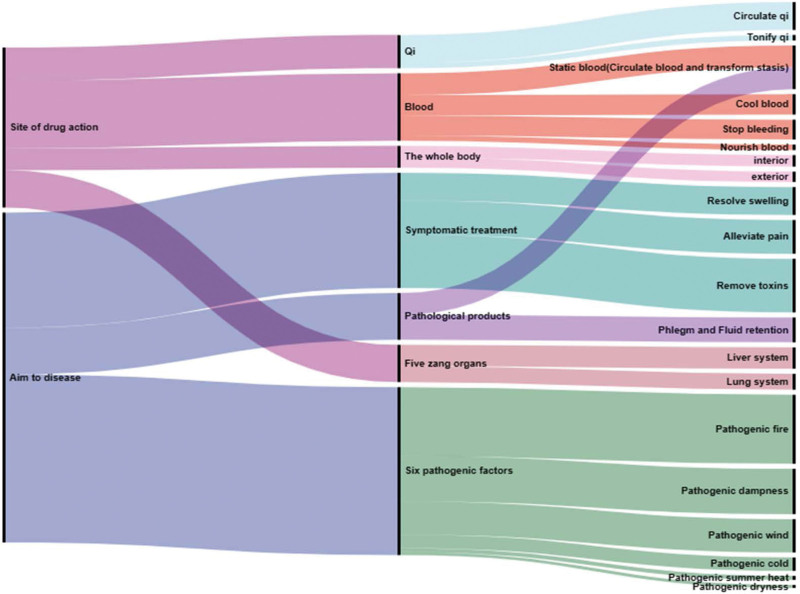
Sankey diagram of herb efficacy. The left side of the figure is the herb function section, which divides the herb efficacy into human-specific and disease-specific. In the middle is the main branch section, which further divides the categories on the left. The right side is the specific branch section, which further subdivides the classification in the middle, and expresses the specific role of the herb in detail.

The efficacies of 367 herbs were analyzed by character decomposition and frequency counting, resulting in a total of 3399 Chinese characters. The median character count was 16. By setting the criterion of key characters as twice the median count, 25 key characters were identified. Based on these key characters, the efficacies of the herbs were systematically verified, and were grouped according to their therapeutic mechanisms and targeted pathogenic factors. Meaningless characters were excluded, and a Sankey diagram was generated based on the results (see Fig. 6). The diagram revealed that potential Chinese medicines for treating Sarcopenia mainly target at diseases caused by fire, dampness, wind, toxins, and stagnant blood, with a focus on promoting blood circulation and adjusting qi and blood.

After the arrangement and combination of the 7 major herbs, 2 new formulas containing 4 major herbs were retrieved from the formulae database. These formulae are Gu Jing Gao, which contains Licorice, Salvia miltiorrhiza, Morus alba bark, and Mulberry leaf, and Da Bu Yan Ling Gao, which contains Licorice, Rhizoma Homalomenae, Salvia miltiorrhiza, and Morus alba bark.

### 3.6. Molecular docking

**Figure 7.**
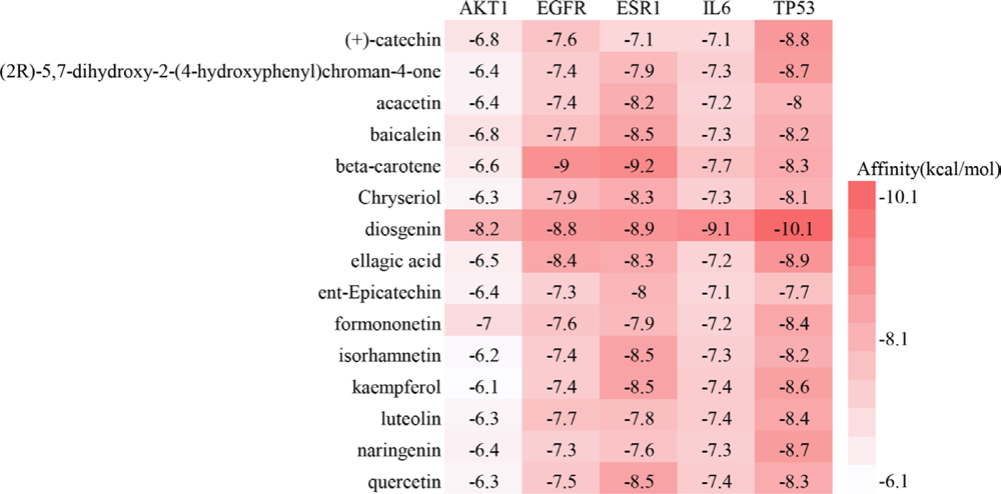
Results of molecular docking. Displays the affinity between the target and the compound, the higher the affinity, the redder the color.

**Figure 8.**
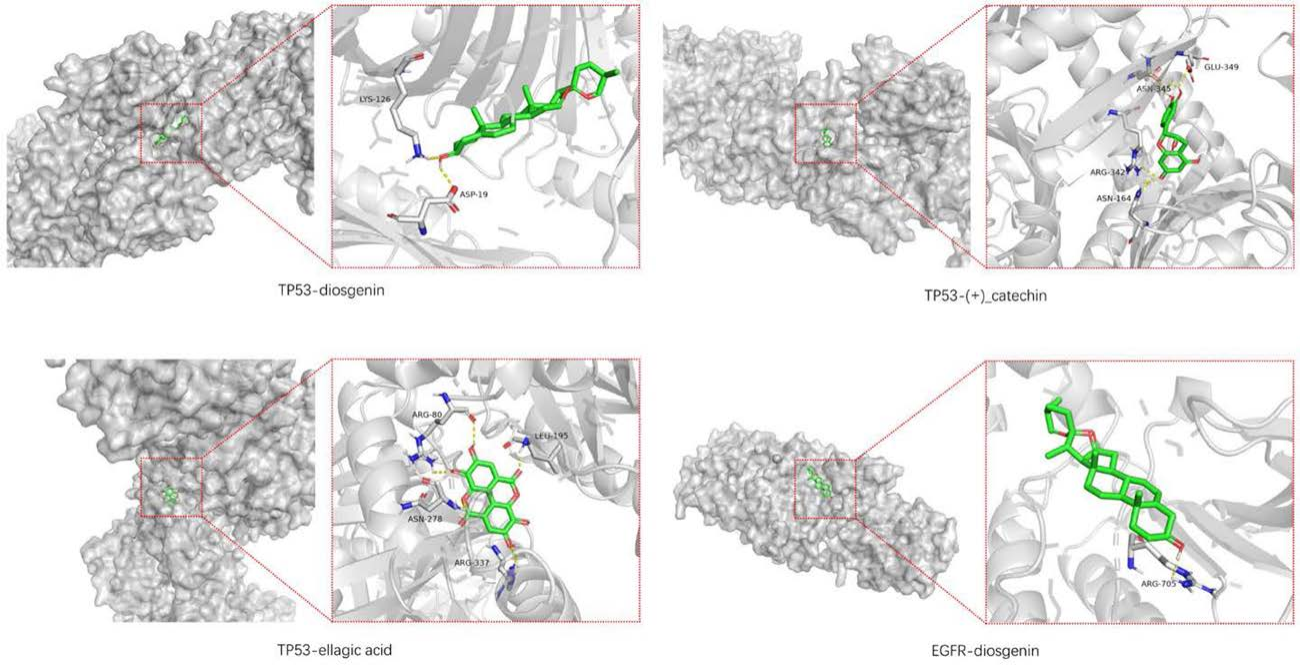
Molecular docking of key targets with key compounds. The yellow lines represent the hydrogen bond interaction force, which is the main force promoting molecule binding with the active site.

Molecular docking was performed between key targets and key compounds, resulting in a total of 75 receptor-ligand complexes. Some of the docking results were visually represented in 3-dimensional displays, as depicted in Figures 7 and 8. Notably, an affinity value of less than −4.25 kcal/mol suggests a potential for ligands and receptors to bind, while an affinity value of less than −5.00 kcal/mol indicates a favorable binding strength. Moreover, an affinity value of less than −7.00 kcal/mol signifies a satisfactory binding strength.^[27]^ In this study, all 75 complexes exhibited an affinity value below −5.00 kcal/mol, with only 13 complexes demonstrating a binding energy greater than −7.00 kcal/mol. This robustly suggests a strong binding relationship between the key target sites and key compounds in the investigation.

## 4. Discussion

### 4.1. Target

This study is based on target-compound network screening, which has identified 5 key targets (ESR1, TP53, IL6, AKT1, and EGFR). ESR1 has been confirmed to be expressed and widely present in human skeletal muscle,^[28]^ and its expression in skeletal muscle can be modulated by endurance training, indicating that ESR1 expression may change in response to functional demands of the muscle.^[29]^

However, there are conflicting views on the role of TP53 in skeletal muscle. Early studies suggested that TP53 is a major driving factor in age-related changes in most cell types and tissues,^[30,31]^ and transgenic mice with increased TP53 activity exhibit premature development of age-related phenotypes, including skeletal muscle atrophy.^[32]^ As a further confirmation, subsequent experiments have shown that TP53 expression in skeletal muscle fibers induces muscle atrophy and is a necessary condition for at least one type of acute muscle atrophy.^[33]^ However, recent research evidence suggests that age-related skeletal muscle atrophy or weakness does not necessarily require TP53 activity in skeletal muscle fibers,^[34]^ and TP53 may actually contribute to maintaining mitochondrial content and function.^[35]^ These findings suggest that TP53 may have a bidirectional regulatory role in skeletal muscle, as sufficient studies indicate that TP53 can participate in pathways that activate cellular repair time and induce cell apoptosis with increased oxidative stress intensity and duration,^[36]^ hinting at the fact that TP53 may be crucial for normal muscle function.

In contrast to historical views, recent research has shown that IL6 levels increase during exercise, indicating a beneficial role of IL6 in exercise capacity.^[37–39]^ However, there is no consensus on the mode of action of IL6. Some theories suggest that IL6 signals in osteoblasts to support osteoclast differentiation and release of bioactive osteocalcin into the circulation, thereby promoting nutrient uptake and breakdown metabolism into muscle fibers in an osteocalcin-dependent manner during exercise, thereby enhancing exercise capacity.^[39]^ Another viewpoint suggests that IL6 is secreted from muscles in response to energy deficiency and breaks down fats to release cellular energy, thereby enhancing muscle energy uptake and temporarily downregulating immune function from the perspective of energy allocation.^[40]^ Despite the lack of consensus, existing theories still support the beneficial effects of IL6 in terms of improving exercise.

Based on the research conducted by Takayoshi Sasako et al in 2022,^[41]^ it has been shown that double knockout of AKT1 and AKT2 in mice results in gradual reduction of skeletal muscle mass, impaired exercise function, systemic insulin sensitivity, as well as decreased bone density and shortened lifespan. This highlights the significance of AKT1 in skeletal muscle and suggests the potential impact of AKT activity on Sarcopenia and lifespan. In terms of mechanism, Natasha Jaiswal et al^[42]^ have validated that AKT acts as a specific intermediate in the insulin or IGF-1 signaling pathway, controlling muscle quality through coordination with FOXO1 and mTORC1 pathways.

EGFR has been demonstrated to play a role in increasing muscle mass or promoting muscle regeneration from various perspectives.^[43,44]^ However, recent research findings indicate that EGFR is not solely responsible for muscle gain, as the use of EGFR inhibitors in mice with muscle dysfunction can partially restore markers of muscle and mitochondrial dysfunction.^[45]^ This suggests that EGFR may have a bidirectional regulatory effect on muscle quality and function.

### 4.2. Compound

The top 3 key compounds obtained in this study are quercetin, kaempferol, and luteolin. Aging and muscle diseases often lead to decreased migration and differentiation of myoblasts, resulting in impaired skeletal muscle function and regeneration.^[46]^ However, quercetin can induce and promote myogenic differentiation through the activation of p-IGF-1R, transcription factor STAT3, and AKT signaling pathways. Moreover, AKT activation has been demonstrated to be a necessary condition for quercetin-induced myogenic differentiation, a fact consistent with the molecular docking results of this study. Dietary supplementation of quercetin has been shown to promote anti-fatigue capacity through its antioxidant ability, glycogen storage, and enhancement of muscle function,^[47]^ serving as an addition to previous studies that have shown significant effects of physical activity and resistance exercise in the treatment of muscle atrophy and improvement of skeletal muscle strength.^[7]^ Quercetin not only enhances muscle function but also strengthens fatigue resistance in patients, thereby improving compliance with exercise therapy and potentially playing a crucial role in the treatment of sarcopenia.

Although there is limited literature on the interaction between naringenin and skeletal muscles, still there is evidence suggests that naringenin can positively regulate the growth and differentiation of skeletal muscles through upregulation of the PI3K, AKT, and MTOR pathways, as well as downregulation of the negative regulatory pathway, Smad pathway, which were involved in skeletal muscle growth,^[48]^ indicating a significant role of naringenin in promoting skeletal muscle growth.

Luteolin, as a common constituent in medicinal formulations, has been proven to possess properties that alleviate muscle atrophy^[49]^ and enhance muscle strength.^[50]^ However, studies solely based on its common constituents are unable to fully elucidate the role of luteolin in medicinal applications. Recent research investigating the effects of luteolin alone on sarcopenia in mice has shown that luteolin exerts inhibitory effects on obesity, inflammation, and protein degradation. Furthermore, the suppression of muscle inflammation by luteolin has been demonstrated to lead to the inhibition of myostatin, indicating its protective effect against sarcopenia in obese individuals.^[51]^

What is noteworthy is that molecular docking studies have shown that diosgenin exhibits the highest binding affinity with TP53. However, despite the promising results, there is a lack of research investigating the impact of the binding between diosgenin and TP53 on skeletal muscle. Studies on diosgenin have demonstrated its ability to significantly increase the diameter and area of skeletal muscle fibers in the thighs of rats, and to induce myoblast fusion, and enhance skeletal muscle through AMPK activation in skeletal muscle cells.^[52]^ Notably, diosgenin possesses bidirectional regulatory capabilities similar to TP53, and thus the results of the docking studies warrant further investigations and academic attention.

### 4.3. Herb

Unexpectedly, licorice, which has traditionally played a supporting role in the composition of TCM formulas, was found to have the highest Degree value among the herbs obtained in this study, surpassing other herbs significantly. The crude water extract of licorice was found to enhance muscle regeneration by inducing myogenic gene expression and downregulating myostatin expression.^[53]^ Moreover, studies on licorice flavonoid oil have shown that this extract can activate AMPK and AMPK-stimulated SIRT1 in muscle cells. Activation of SIRT1, in turn, can inhibit FoxO in the muscle degradation system. Therefore, ingestion of licorice flavonoid oil not only promotes muscle cell growth, but also inhibits muscle cell breakdown.^[54,55]^ Based on the positive results of 2 double-blind trials expressing the improvement of muscle function in middle-aged and elderly individuals with the intake of licorice flavonoid oil,^[55,56]^ it can be inferred that licorice may have a unique therapeutic effect on sarcopenia.

TCM does not have a definitive definition for sarcopenia, but the definition of Wei syndrome (a type of disease characterized by slow and weak limbs, inability to move freely, or muscle atrophy) aligns well with the diagnostic criteria for sarcopenia. Based on the understanding of Wei syndrome in Chinese medicine, the main causes are attributed to pathogenic factors such as warm toxin, dampness-heat, and blood stasis due to trauma or impact. Combined with the analysis of potential herbs from the 3.5 items, it is found that the herbs commonly used for treating sarcopenia in Chinese medicine are mostly cold and warm in nature, with specific effects on detoxification, heat, dampness, and blood stasis. According to the TCM theory of “treating cold with heat, and heat with cold,” the effects of the herbs obtained match well with the pathogenic factors of Wei syndrome. Moreover, according to the TCM belief, the organs most closely related to the pathological changes of Wei syndrome are liver, spleen, stomach, and lungs, which also highly correspond to the results of herb analysis from the 3.5 items.

From the perspective of Wei syndrome, the effective prescriptions provided by TCM contain major herbs identified from the 3.4 items. However, a mature prescription typically includes only 2 to 3 major herbs. In order to enhance the efficacy of prescriptions on the targets, this study conducted permutation and combination among the 7 major herbs, and searched the prescription database to identify 2 new prescriptions. Theoretically, the 2 prescriptions derived from the research results contain more compounds with potential effects on sarcopenia compared to existing prescriptions, and may have more significant therapeutic effects in treating sarcopenia when compared to traditional prescriptions.

### 4.4. Molecular docking

In this study, both diosgenin and TP53 exhibit a pronounced affinity for their respective targets, with the highest affinity between diosgenin and TP53 reaching −10.1 kcal/mol. The investigation^[57]^ indicates that in cancer patients experiencing skeletal muscle loss, cancer cell secretions, upon transfer into muscle cells, selectively inhibit the expression of TP53. However, upon restoration of TP53 function, mitochondrial myopathology is alleviated, and damaged running capacity is rescued. We have grounds to believe that the normal expression of TP53 plays a vital role in maintaining mitochondrial homeostasis and, furthermore, that TP53 can decelerate muscle wasting caused by mitochondrial factors, thereby preserving normal physiological processes. Although research on the combined effects of diosgenin and TP53 on skeletal muscle remains incomplete, the regulatory capacity of diosgenin on TP53 has already been confirmed.^[58]^

Regarding the treatment of sarcopenia, these discoveries hold significant importance. In addition to promoting muscle growth, limiting muscle loss is a crucial aspect when confronting sarcopenia. TP53 is often regarded as a cancer inhibitor due to its role in apoptosis. However, the above-mentioned research indicates that TP53 impact on apoptosis might not be unidirectional reduction; instead, TP53 expression acts more like a “governor,” restricting excessive proliferation and rapid loss, thus restoring cells to their normal physiological processes.

### 4.5. Strengths and limitations

The strength of this study lies in the utilization of emerging methodologies such as network pharmacology and data mining to screen a plethora of herbs with the potential to treat sarcopenia, thus economizing both time and resources for future research. Traditional Chinese medicine, being an experienced medical system that has long employed a holistic approach to combined drug therapy, possesses an unparalleled advantage in the face of diseases lacking effective treatment methods. While this study provides a reference for similar research, it must be acknowledged that our analysis of the relationships between targets, compounds, and herbs remains largely theoretical. The discussion about the content and proportion of compounds in each herb and their therapeutic effects is yet to be adequately addressed. Furthermore, due to prudent safety considerations, drugs with organ toxicity were not included in this study, which may result in the oversight of potential drug combinations. Nevertheless, no relevant reports have been found thus far, and we will continue to monitor any relevant developments.

## 5. Conclusion

This study is based on the selection of sarcopenia-related targets from multiple databases, as well as the screening of compounds and herbs from the TCMSP platform to construct a network diagram. Potential targets, compounds, and herbs that may contribute to the treatment of sarcopenia were screened from the network, and molecular docking verification was performed on key targets and compounds. Through data mining techniques from the perspective of TCM, potential herb analysis was conducted, and novel formulas were explored. These findings provide pharmacological validation for the potential of TCM in treating sarcopenia and offer new insights for the selection of TCM for sarcopenia treatment.

## Acknowledgments

In this study, we express our profound gratitude to the many individuals and resources involved. Their support and contributions have been indispensable in facilitating the smooth progress and achievements of our research. Firstly, we extend our appreciation for the existence of public databases. These databases have provided us with abundant data resources, offering robust support to our research. The diligent work and efforts of the developers and maintainers of these public databases have made significant contributions to scientific research and the academic community. Furthermore, we acknowledge the support of software. In our research, we have utilized various advanced software tools and techniques, which have provided us with powerful computational and analytical capabilities. We are grateful to the developers of these software, whose dedication and innovation have provided invaluable technical support to our research. Lastly, we express sincere gratitude to the participants who have contributed to our study. Their valuable feedback, suggestions, and assistance at different stages of the research have played a crucial role in the successful completion of our study. While we are unable to list all individuals and resources here, we wish to extend heartfelt thanks to everyone who has supported and assisted our research. Your contributions have made our research more rigorous, comprehensive, and valuable. Once again, our deepest appreciation to all those who have supported us! Thank you!

## Author contributions

**Conceptualization:** Changwen Zhou.

**Data curation:** Hongzhong Ma, Ce Liu.

**Methodology:** Changwen Zhou, Lixue Yang.

**Supervision:** Lixue Yang.

**Visualization:** Hongzhong Ma, Lixue Yang.

**Writing – original draft:** Changwen Zhou, Ce Liu.

**Writing – review & editing:** Hongzhong Ma, Lixue Yang.
